# Transcutaneous spinal direct current stimulation induces lasting fatigue resistance and enhances explosive vertical jump performance

**DOI:** 10.1371/journal.pone.0173846

**Published:** 2017-04-05

**Authors:** Helen R. Berry, Rothwelle J. Tate, Bernard A. Conway

**Affiliations:** 1 Centre for Excellence in Rehabilitation Research, Department of Biomedical Engineering, University of Strathclyde, Glasgow, United Kingdom; 2 Strathclyde Institute for Pharmacy and Biomedical Sciences, University of Strathclyde, Glasgow, United Kingdom; University of Ottawa, CANADA

## Abstract

Transcutaneous spinal direct current stimulation (tsDCS) is a non-invasive neuromodulatory intervention that has been shown to modify excitability in spinal and supraspinal circuits in animals and humans. Our objective in this study was to explore the functional neuromodulatory potential of tsDCS by examining its immediate and lasting effects over the repeated performance of a whole body maximal exercise in healthy volunteers. Using a double-blind, randomized, crossover, sham-controlled design we investigated the effects of 15 min of anodal tsDCS on repeated vertical countermovement jump (VCJ) performance at 0, 20, 60, and 180 minutes post-stimulation. Measurements of peak and take-off velocity, vertical displacement, peak power and work done during countermovement and push-off VCJ phases were derived from changes in vertical ground reaction force (12 performance parameters) in 12 healthy participants. The magnitude and direction of change in VCJ performance from pre- to post-stimulation differed significantly between sham and active tsDCS for 7 of the 12 VCJ performance measures (P < 0.05). These differences comprised of a post-sham fatigue in VCJ displacement/work done, peak to peak power and take-off velocity, and a resilience to this fatigue effect post-active tsDCS. In addition there was also an enhancement of countermovement performance and total work done (*P* < 0.05). These changes did not vary across repeated VCJ performances over time post-tsDCS (P > 0.05). Our original findings demonstrate that one single session of anodal tsDCS in healthy subjects can prevent fatigue and maintain or enhance different aspects of whole body explosive motor power over repeated sets of VCJs performed over a period of three hours. The observed effects are discussed in relation to alterations in central fatigue mechanisms, muscle contraction mode during jump execution and changes in spinal cord excitability. These findings have important implications for power endurance sport performance and for neuromotor rehabilitation.

## Introduction

Transcutaneous spinal direct current stimulation (tsDCS) is a relatively new approach to neuromodulation, with increasing literature since 2008 providing evidence of short lasting modulatory effects on spinal and supraspinal function. To date, researchers have reported modulation of segmental spinal reflex behaviours [1–4], corticospinal excitability [5] and somatosensory pathway conduction [4, 6] in humans. Research on anaesthetised rodents has shown spinal DCS to influence supraspinal activity [7, 8] and to modulate muscle force production, reflexive actions and locomotor activity [9, 10]. Although these animal studies used invasive electrode placements, they do raise the possibility that tsDCS may modulate functional motor outcomes in humans.

Research on transcranial direct current stimulation (tcDCS) indicates that its effects are dependent on the orientation of the underlying neural compartments relative to the direction of current flow [11]. Similarly, tsDCS-evoked facilitation or inhibition of spinal neural pathways and circuits are also reported to be polarity dependent and to persist post-stimulation [12]. In rodent studies, anodal tsDCS is reported to depress cortically elicited muscle contractions during stimulation, but potentiate them post-stimulation, with the opposite true for cathodal tsDCS [9]. Polarity effects on target pathways will also depend on the site of stimulation (e.g. cervical or lumbosacral) [5, 12]. As a method of neuromodulation tsDCS is non-invasive, well tolerated by subjects, and there have been no adverse events reported following single session tsDCS interventions [6, 12].

The spinal cord is an attractive target for neuromodulation. Segmental and intersegmental circuits integrate and process multisensory and supraspinal information to refine and adapt the motor output patterns needed to coordinate and successfully perform functional motor tasks (e.g. standing, running, walking or jumping). Repeated practice and training constantly stimulate adaptation and plasticity in spinal and supraspinal pathways as a prerequisite for motor skill learning [13]. TcDCS neuromodulation has also been found to enhance motor skill learning via similar neuroplastic mechanisms as practice mediated plasticity [14]. By targeting the integrative neural pathways and circuits of the spinal cord, anodal tsDCS may therefore have the potential to exert positive neuromodulatory actions on circuits that influence coordinated, whole body motor performance. These effects may be beneficial in sports or during rehabilitation following brain or spinal cord injury. However, at the present time it is not known whether the application of lumbosacral tsDCS translates to any measurable or lasting effects on motor power or voluntary motor performance in humans.

Therefore the aim of this study was to determine if tsDCS applied over the lumbosacral cord could change the functional performance of a human whole body motor task. The motor task studied here is the vertical countermovement jump (VCJ) with arm swing which is a powerful, explosive plyometric exercise that requires the coordination of head, neck, trunk, and all four limbs. VCJ performance is used as a measure of whole body power in athletics [15–17] and can be characterised through a range of measures derived from changes in the vertical ground reaction force (GRF) generated as a person prepares and executes the jump [16–18]. This motor task was chosen to maximise our chances of detecting any tsDCS effects on motor power during an unconstrained task comprising repeated maximal eccentric and concentric muscle activations. Although this particular motor task is not appropriate in a neurorehabilitation setting, changes in VCJ performance after tsDCS may indicate the rehabilitative potential of tsDCS for modulating motor control circuits including those involved in sitting, standing and falls prevention. In this exploratory pilot study, we investigated the influence of 15 minutes of sham and active anodal tsDCS (double blind, cross over design) on repeated maximal effort VCJ performance in healthy human volunteers over a period of three hours.

## Methods

### Subjects and ethical approval

Following approval by the University of Strathclyde Research Ethics Committee and in accordance with the declaration of Helsinki, 12 (3 female) normally active, healthy volunteers (*M* ± *SD*: age 29 ± 11 years and BMI 23 ± 2) were recruited from the University campus between July 2014 and February 2015 and gave their fully informed written consent to participate in the study. Six of these participants (1 female) had experienced one 15 min tsDCS application 4–6 months previously for a separate investigation. The individual shown performing the VCJ manoeuvre in the supporting video (S1 Video) has given written informed consent (as outlined in PLOS consent form) to publish this video.

All participants attended two separate test sessions in order to investigate the effects of sham and active anodal tsDCS on VCJ performance. The minimum time interval between sessions was one week. Participants were asked to avoid any intensive or unaccustomed physical activity in the week preceding the tests and to avoid consuming any food or caffeinated beverages within the two hours prior to the start of each session. Otherwise, no dietary or fluid intake restrictions were applied over the course of the study. Body mass and height were recorded for all subjects during the initial consenting visit and prior to testing. Subjects were also familiarised with the tsDCS intervention and VCJ protocol and were given the opportunity to practice VCJ performance.

### Vertical countermovement jump

**Fig 1:**
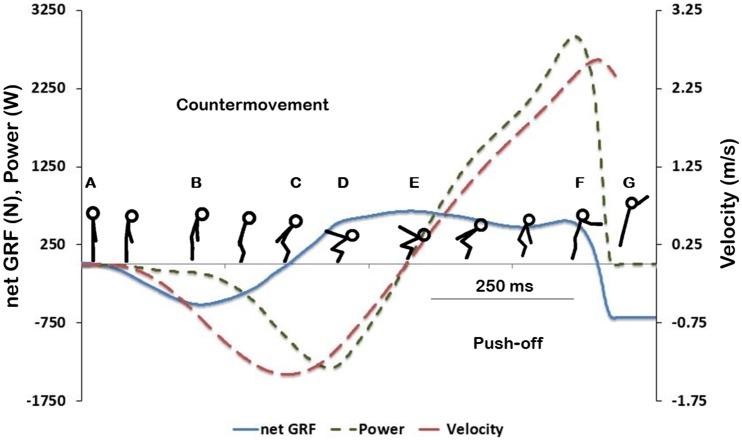
Net vertical Ground Reaction Force (GRF), power and velocity time curves during a representative trial of a Vertical Countermovement Jump (VCJ). The countermovement (CM) phase of the VCJ (A—E) starts with a short period of unweighting (A—B). Lower limb muscles activate to brake as the body accelerates downwards (B—C) and GRF returns to zero at peak downwards velocity (C). The arms raise backwards, as the body decelerates (C—E) to a momentary halt at transition point (E) where velocity and power have returned to zero. The push-off phase of the jump is immediately initiated as the body segments extend and accelerate upwards to take-off (E—G), which occurs shortly after peak power and velocity have been reached respectively and the GRF curve returns to zero.

Standing on an instrumented platform (detailed below), participants performed sets of 5 maximal effort VCJs immediately prior to and at 0, 20, 60, and 180 minutes after application of sham or active anodal tsDCS (25 VCJs per test session, 50 VCJs in total). Rest periods of 20 seconds were given between each VCJ effort, approximating to a 1:20 work to rest ratio. This was determined to be sufficient to regenerate the immediate ATP phosphocreatine energy system between each VCJ and to avoid peripheral muscle fatigue effects. Each of the five VCJ sets were preceded with a moderately paced 2 min warm up comprising stepping on and off a 15 cm stepper (Reebok, Adidas, Herzogenaurach, Germany). Participants were instructed to jump with maximal effort and to use a consistent jump technique throughout. Volunteers initiated their VCJ with a downward countermovement from a standing position and, using their arms for leverage, jumped up from the force plate with maximal effort. See Fig 1 and S1 Video

### Data collection

Vertical ground reaction forces (GRF) were recorded using a force plate (Kistler Type 9865, Kistler Instrumente AG, Winterthur, Switzerland) connected to a Vicon Data Station 612 (Vicon Motion Systems, Oxford Metrics LTDA, Oxford, UK) using Vicon’s proprietary software: Nexus version 1.8.5. GRF was sampled and recorded at 1 kHz. Data were initially processed offline using Microsoft Excel 2010: GRF values were filtered using a 2nd order low pass Butterworth filter, with a cut off frequency of 50Hz. Instantaneous impulse (Ns), velocity (V), and power relative to body mass (W∙kg^-1^), were calculated from the filtered GRF data, from when the jumper was stationary through to the instant of take-off, using the following equations:

$$N_{s}=\frac{GRF_{t2}-GRF_{t1}}{2X}\overset{X}{\underset{k=1}{\sum }}\left(\left(N_{w}-GRF_{t+k}\right)+\left(N_{w}-GRF_{t}\right)\right)$$

$$v=\frac{N_{s}}{N_{m}}$$

$$w=\frac{GRF_{t}\times v}{N_{m}}$$

Where: *Ns* = Impulse in Newtons, *GRF*_*t*_ = Vertical ground reaction force in Newtons at time, in seconds *t*; *t*_*1*_ = time at start of the jump; *t*_*2*_ = time at end of the jump; *Nw* = Body weight in Newtons; *v* = velocity in m∙sec^-1^; *w* = normalised instantaneous power in W∙Kg^-1^.

#### Outcome variables

To provide a comprehensive analysis of performance, 12 VCJ performance outcome variables [16, 19] (detailed in Table 1) were determined at key points during the VCJ. These included the unweighting and braking phases of countermovement (CM), transition point, and the push-off phase of the VCJ, as illustrated in Fig 1 and detailed in Table 1. Table 1 also provides average baseline values for each measurement.

**Table 1:** Vertical Countermovement Jump (VCJ) phases, outcome variables and baseline raw values for each.

	Pre-tsDCS	Unweighting	Braking	Transition	Push-off
	Mean ± SD	A—B	B—E	E	E—G
Unweighting GRF (N∙kg^-1^)	5.8 ± 1.6	GRF			
CM velocity (m∙s^-1^)	1.1 ± 0.2	peak velocity			
CM power (W∙kg^-1^)	14.6 ± 4.0	peak power			
CM displacement (cm)	33.1 ± 6.5	Vertical displacement			
Transition GRF (N∙kg^-1^)	10.2 ± 4.2			GRF	
Push-off GRF (N∙kg^-1^)	12.9 ± 3.7				peak GRF
Push-off velocity (m∙s^-1^)	2.6 ± 0.2				peak velocity
Push-off power (W∙kg^-1^)	48.4 ± 6.2				peak power
Push-off work done (J∙kg^-1^)	7.4 ± 0.9				work done
Peak to peak power (W∙kg^-1^)	63.0 ± 9.1	peak to peak power			
Total work done (J∙kg^-1^)	10.7 ± 1.4	total work done			
Take-off velocity (m∙s^-1^)	2.4 ± 0.2	final velocity			

Variables of interest during each phase of the vertical countermovement jump (VCJ). Phases A—G are illustrated and described in Fig 1. Data are mean ± SD for all raw pre-tsDCS values: CM; countermovement, GRF; vertical ground reaction force calculated net of bodyweight, Velocity and power were calculated from the integral of the GRF—time curve and the velocity—time curve. Vertical displacement and work done were calculated as the areas under the velocity and power curves respectively.

The filtered data were imported into Spike2 v 8 software (Cambridge Electronic Design Ltd, Cambridge, UK) for ease of visualisation and further signal processing through a customised software script where the areas under the velocity—time, and power—time curves were calculated to determine vertical displacement and work done during each VCJ phase. Work done was normalised to body mass. During CM, changes in vertical displacement and work done were equivalent, therefore only data for changes in vertical displacement are presented here. Peak values for each variable were determined as the maximum positive or negative value achieved during each phase. Unweighting was determined as the maximum negative GRF value during CM (B, Fig 1). All negative values were inverted. Transition GRF was determined at the end of CM where downward velocity had returned to zero (E, Fig 1). For each of the 12 variables, the average value over all 5 VCJ efforts within each set was used for analysis.

### tsDCS intervention

#### Electrode montage

**Fig 2:**
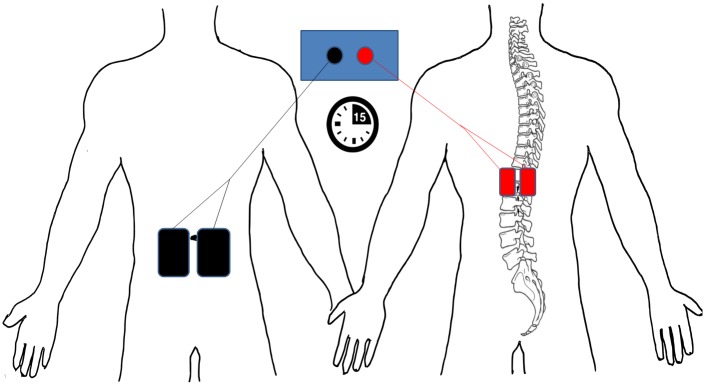
Electrode montage for tsDCS. The bifurcated anode (red) is placed centrally and paravertebrally over T11, T12 thoracic vertebrae and the bifurcated cathode (black) is placed centrally and lateral to the umbilicus. 2.5 mA of anodal direct current was applied for 15 minutes, providing 0.0833 mA/cm^2^ current density and 0.075 C/cm^2^ of total current applied.

A pair of commercially available stainless steel knit fabric self-adhesive 3 x 5 cm electrodes (Pals, Axelgaard, Lystrup, Denmark) were placed on the skin 0.5 cm paravertabrally and centrally over the T11 and T12 vertebrae thereby overlying the lumbosacral enlargement. This electrode pair was coupled together to function as one active 30 cm^2^ electrode. A second pair of 7.5 x 13 cm electrodes, coupled to function as one 195 cm^2^ dispersive electrode (Valutrode, Axelgaard, Lystrup, Denmark), was placed longitudinally on the abdomen at each side of the umbilicus [20] (Fig 2).

This trans-abdominal electrode montage conforms with layouts identified through computer modelling as delivering a higher, more focused current density over the lumbosacral cord when compared to the more common spine to shoulder or arm tsDCS montages used in previous human tsDCS studies [21]. Electrode positions were photographed and reproduced for each test session. The use of self-adhesive conductive fabric electrodes for tsDCS application allowed subjects to perform VCJs without restriction or discomfort.

The order and delivery of sham and active tsDCS were randomised and double blinded, with stimulator codes being allocated to each condition and subject prior to the start of the study by an independent researcher. Participants lay supine on a comfortable treatment couch during stimulation and were instructed to lie quietly throughout. Polarization was achieved through 15 min of 2.5 mA anodal tsDCS (the polarity was determined by the paravertebral electrode, see Fig 2) delivered using a DC stimulator (NeuroConn, Ilmenau, Germany). This provided a current density of 0.0833 mA/cm^2^, and total delivered current of 0.075 C/cm^2^ which is a common tsDCS current paradigm and well below the threshold for potential tissue damage [6, 22].

For both sham and active tsDCS, stimulation was ramped up from 0 to 2.5 mA over a 10 s period and similarly ramped down after 900 s to minimise sensation at onset and offset of stimulation. The stimulator sham tsDCS mode (inbuilt ‘Study Mode’ protocol) maintained the 2.5 mA current for only 30 seconds and was then followed by 110 μA pulses applied for 15 ms every 550 ms thereafter, corresponding to a mean current of 1.7 μA. This provided similar skin sensations to those felt during active stimulation, but with negligible biological effects [1].

### Data analysis

All data were calculated and analysed as ratios to pre-tsDCS baseline values (Δ). For simplicity, results are described as percentage values rather than ratios. Significance was determined at *P* ≤ 0.05. The data are the results of a pilot study, and therefore power and sample size were not determined a priori. Instead the effect size, *d*, was calculated post hoc for significant main effects [23]. Statistical analyses were performed using Minitab (version 17.0, Minitab, State College, PA, USA).

Our hypothesis was that Δ VCJ performance would differ between sham and active tsDCS and that these changes would increase or decrease following each subsequent, additional set of VCJs performed within a 180 min period post-stimulation. To test this we performed a general linear mixed model ANOVA with each of the 12 dependent Δ VCJ variables [24]. Controlling for random subject variation, anodal tsDCS condition (sham 0 or active 1) and time of post-tsDCS VCJ repeat (0, 20, 60 and 180 min) were the independent factors and included a tsDCS condition by time interactive term. We tested all data for normality of distribution (Anderson-Darling) before analysis and, where necessary, transformed the data using a Johnson transformation. When differences were significant, post hoc comparisons (Tukey method) were performed to quantify these differences and the substantive effect size [25] was determined from this by calculating Cohen’s *d* [23]. We then used t-tests to determine the magnitude and direction of these changes relative to baseline.

## Results

Participants reported that they were not able to recognise the difference between application of sham or anodal tsDCS. Perception of the stimulation was limited to some participants being aware of a slight itching or tingling below the electrodes. In some instances mild reddening of the skin occurred below the electrodes but this was only apparent at the end of the test session and quickly resolved after the electrodes had been removed.

**Fig 3:**
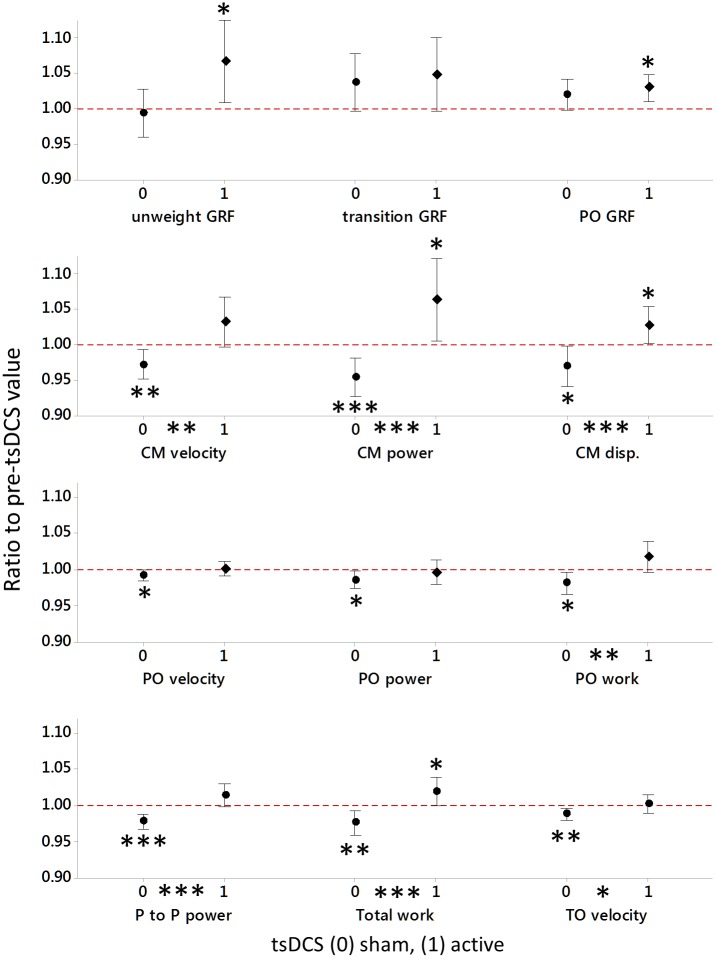
Mean change relative to baseline in VCJ performance after sham and active anodal tsDCS. Data are the mean ratios to pre-tsDCS values and 95% CIs for 12 subjects pooled over all four repeated VCJ sets (0–180 min), post-sham (0) and active (1) tsDCS stimulation. Significant differences between sham and active tsDCS are indicated on the X axis. Mean changes were different from baseline where the 95% CI did not cross the red dotted line (baseline value, 1.00). Significance was indicated as: *P ≤ 0.05, **P ≤ 0.005 and ***P ≤.0.001. CM; countermovement, disp; downward vertical displacement, GRF; vertical ground reaction force, PO; push-off, P to P; peak to peak, trans; transition, unweight; unweighting.

For 7 of the 12 ΔVCJ performance parameters, there was a significant main effect of tsDCS (sham 0 vs active 1) and this effect was irrespective when the VCJ sets were repeated post-sham and anodal tsDCS, or of individual variability in response to stimulation (see Fig 3 and Table 2). For these 7 ΔVCJ performance parameters, the substantive effect of tsDCS, determined by effect size [25], was largest for the differences in changes in CM velocity, followed by changes in peak to peak power, CM power, total work, CM displacement and push-off work, all with moderate effects. The smallest significant effect was on change in take-off velocity (Table 3).

**Table 2:** Outcome from general linear mixed model ANOVA.

		tsDCS	time	tsDCS*time	Subject
	DF: error 72	1	3	3	11
unweighting GRF	*F*	2.62	0.37	0.75	1.23
	*P*	0.110	0.778	0.528	0.284
CM velocity	*F*	9.62	0.66	1.25	3.09
	*P*	0.003	0.578	0.300	0.002
CM power	*F*	11.33	0.65	1.62	3.45
	*P*	0.001	0.586	0.193	0.001
CM displacement	*F*	12.38	0.30	0.85	3.33
	*P*	0.001	0.826	0.469	0.001
Transition GRF	*F*	0.02	0.78	0.62	5.47
	*P*	0.900	0.511	0.606	0.000
Push-off GRF	*F*	0.86	0.50	0.34	9.59
	*P*	0.356	0.685	0.799	0.000
Push-off velocity	*F*	2.85	0.41	0.33	3.9
	*P*	0.096	0.746	0.803	0.000
Push-off power	*F*	1.74	0.41	0.48	5.76
	*P*	0.191	0.750	0.700	0.000
Push-off work	*F*	8.49	0.12	0.19	2.50
	*P*	0.005	0.950	0.950	0.010
Peak to peak power	*F*	16.88	0.21	1.00	3.93
	*P*	0.000	0.891	0.400	0.000
Total work	*F*	12.95	0.14	0.29	2.12
	*P*	0.001	0.938	0.836	0.029
Take-off velocity	*F*	4.48	0.45	0.34	3.27
	*P*	0.038	0.718	0.800	0.001

N = 12, numerator degrees of freedom (DF) are given below each main effect and interaction term. Denominator DF is the error, 72. The F-ratio (*F*) and P-values (*P*) are given for each of the 12 ΔVCJ performance dependent variables. CM; countermovement, GRF; vertical ground reaction force net of bodyweight. Time refers to the time at which each set of VCJs were successively repeated post-sham and active anodal tsDCS.

**Table 3:** Substantive effects of tsDCS on changes in VCJ performance outcomes between sham and active tsDCS.

	Active Δ	Sham Δ	Diff.	95% CI	*P*	*d*	Effect size
CM velocity	3.3%	- 2.7%	6.0%	2.5, 9.5	0.003	1.94	Large
CM power	6.7%	- 4.4%	11.1%	5.8, 16.5	0.001	0.69	Medium
CM displacement	2.9%	- 3.1%	6.0%	2.6, 9.4	0.001	0.63	Medium
Push-off work	1.6%	- 2.0%	3.6%	1.2, 6.1	0.005	0.55	Medium
Peak to peak power	1.3%	- 2.3%	3.6%	2.0, 5.2	0.000	0.76	Medium
Total work	2.0%	- 2.5%	4.4%	2.0, 6.9	0.001	0.68	Medium
Take-off velocity	0.0%	- 1.3%	1.4%	0.0, 2.8	0.038	0.39	Small

The difference (Diff.) in mean change (Δ) in VCJ determined between sham and active tsDCS from post hoc Tukey tests. (See Fig 3 for 95% CIs of mean changes). Effect sizes, *d* were calculated according to Cohen [23]. Significance at *P* ≤ 0.05.

The effects of tsDCS were manifest post-sham as an overall fatigue in VCJ performance, evidenced by significant reductions from baseline in CM velocity (*t* (47) = -2.87, *P* = 0.006), peak to peak power (*t* (47) = -4.76, *P* < 0.001), CM power (*t* (47) = -3.49, *P* = 0.001), total work done (*t* (47) = -2.99, *P* = 0.004), CM vertical displacement/work done (*t* (47) = -2.27, *P* = 0.028), push-off work done (*t* (47) = -2.52, *P* = 0.015), and take-off velocity (*t* (47) = -3.13, *P* = 0.003) (Fig 3 and Table 3). In addition, although not statistically different from changes observed after anodal tsDCS, there was a clear fatigue in push-off velocity (*t* (47) = -2.36, *P* = 0.022) and push-off power (*t* (47) = -2.61, *P* = 0.012) relative to baseline value (Fig 3).

**Fig 4:**
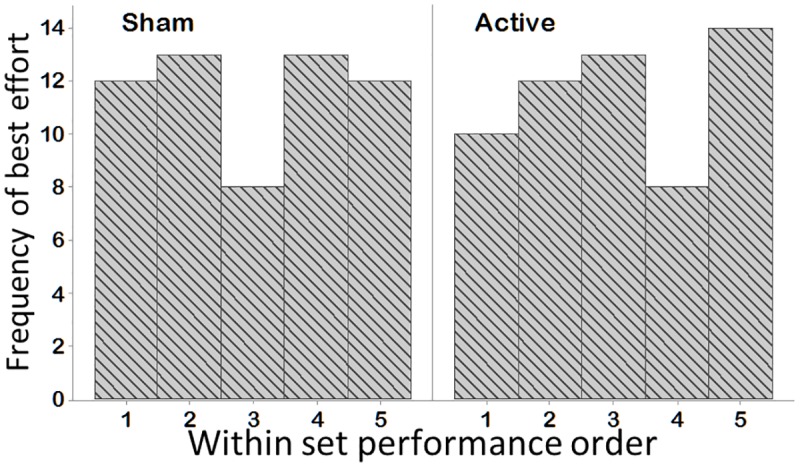
VCJ effort with the highest peak to peak power during each set of 5 maximal VCJ efforts. Over all 12 subjects and all test sessions, the best VCJ effort, determined as the VCJ with the highest peak to peak power was most frequently achieved during the second and fourth attempts in the sham tsDCS session and the fifth attempt during the active tsDCS session.

Importantly, there was no evidence of short term within-set performance fatigue after either sham or active tsDCS: the VCJ with the highest peak to peak power in each set of 5 VCJs was evenly distributed across each of the five efforts: during sham tsDCS, the second and fourth efforts of each set were most commonly the highest value and during active tsDCS, the fifth effort in each set was most commonly the highest (Fig 4).

In response to active anodal tsDCS, there was a preservation of VCJ performance, evidenced by a maintenance in CM velocity (*t* (47) = 1.85, *P* = 0.070), peak to peak power (*t* (47) = 1.67, *P* = 0.101), push-off work (*t* (47) = 1.58, *P* = 0.121), and take-off velocity (*t* (47) = 0.18, *P* = 0.855) at baseline value. Interestingly, there was also a post-anodal tsDCS increase in CM power (*t* (47) = 2.26, *P* = 0.028), CM displacement/work done (*t* (47) = 2.18, *P* = 0.034) and total work done (*t* (47) = 2.01, *P* = 0.050) above baseline (Fig 3 and Table 3). In addition, although not statistically different to changes after sham tsDCS, there was also an increase in unweighting GRF (*t* (47) = 2.39, *P* = 0.021) and push-off GRF (*t* (47) = 3.10, *P* = 0.003) above baseline value after active anodal tsDCS (Fig 3).

**Fig 5:**
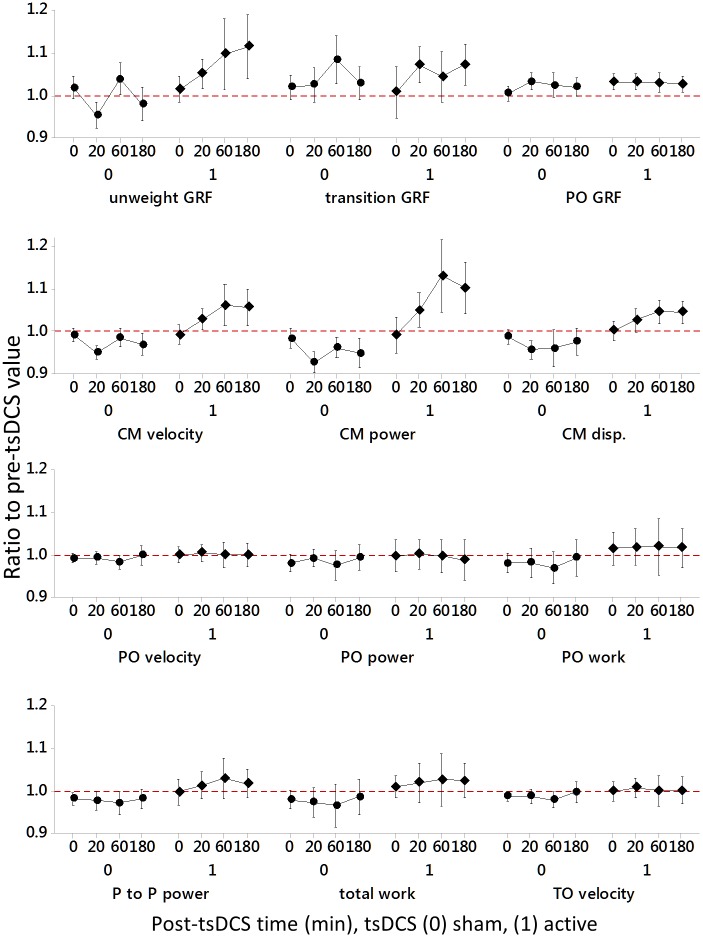
Changes relative to baseline in 12 VCJ performance outcomes performed at four successive time points following sham and active tsDCS. Data were calculated as ratios to pre-tsDCS values, indicated by the red dotted horizontal line at 1, for four successive VCJ sets repeated from 0–180 min following sham (0) and active (1) tsDCS stimulation. Changes in all 12 VCJ performance outcomes were stable over time post-tsDCS (P > 0.05).

As illustrated in Fig 5, there was no main effect of VCJ performance time on changes in all 12 VCJ performance parameters, and no significant interaction between tsDCS condition and time (Table 2 and Fig 5). This tells us that the changes in VCJ performance that occurred after both sham and active tsDCS conditions were stable from the first set of VCJs performed immediately following tsDCS until the final set performed 180 min later. In addition, although the mean changes in VCJ performance differed between tsDCS conditions, there was significant variation between individual subjects in their response to tsDCS for 11 of the 12 ΔVCJ performance outcomes (Table 2). This tells us that there were important factors that led to differences in responsiveness to tsDCS between individual participants that we have not accounted for here.

## Discussion

The aim of this study was to examine the effect of 15 min of sham and active tsDCS on VCJ performance over a period of three hours. Our results provide the first evidence of anodal tsDCS exerting a sustained influence on whole body motor power during the repeated performance of an explosive gross motor task in healthy subjects.

The results presented here demonstrate a clear difference in the magnitude and direction of changes in VCJ performance after sham and active anodal tsDCS. The key finding of this study is that, after sham tsDCS there was a fatigue in VCJ performance apparent over a wide range of outcome measures that was prevented following 15 minutes of 2.5 mA anodal tsDCS conditioning. These changes in performance did not vary significantly over the entire 180 minute test period. This infers that for sham tsDCS, the fatigue process was perpetuated by each subsequent VCJ set performed, whereas anodal tsDCS was able to prevent this fatigue process from engaging at any point over the repeated VCJ sets. However, at the present time, and without the benefit of further detailed physiological investigation, we are unable to state which specific physiological and neuroplastic mechanisms were influenced by anodal tsDCS and therefore can only speculate here based on our observations and the neurophysiological findings of previous tsDCS studies.

Performing repeated maximal effort whole body exercise, leads to central fatigue that diminishes performance output [26, 27]. Repeated performance of stretch-shorten types of exercise also leads to long lasting muscular and neuromuscular fatigue, due, in part, to a combination of eccentric muscle tissue damage, reduced reflex sensitivity and reduced gain in spinal circuitry [26, 28]. The VCJs performed here comprised of repeated cycles of maximal extensor muscle stretch-shortening during the CM, push-off and landing phases of the jump. However, performance within each VCJ set (5 maximal effort jumps performed within an approximate 1 minute time window) did not deteriorate from the first to the fifth repetition (Fig 4) and so it is unlikely that peripheral muscular effects were a significant contributing factor. Accordingly, it seems appropriate to propose that the fatigue observed in VCJ performance post-sham tsDCS condition is of central origin and that it outlasted the 180 minute test period.

The lasting fatigue protection after anodal tsDCS may be due to multiple factors. Localised anodal tsDCS-induced tissue effects may arise during stimulation, but are most likely to influence the paraspinal muscles directly below the stimulating electrodes. Local effects could include an increased local blood flow, changes in metabolism, temperature and potentially alterations in biomechanical properties such as elasticity and stiffness. However, such effects would be transitory and short-lived, persisting for only 8–10 minutes post- stimulation [29]. Accordingly, it would be difficult to envisage how such changes could affect VCJ performance across the 180 minutes post-stimulation assessment period. It is more likely that anodal tsDCS exerted modulatory effects on central fatigue mechanisms.

The sustained fatigue in CM performance observed here after sham tsDCS, evidenced by reductions in CM downward displacement and power, was not only abolished after active tsDCS, but CM performance was considerably enhanced. This would suggest that tsDCS exerted effects on central mechanisms affecting the development of a fatigued state as well as potentiating motor output. The CM and landing phases of the VCJ are dominated by powerful eccentric contractions of leg extensor muscles. Interestingly, Duchateau & Enoka [30] recently observed that spinal and corticospinal excitability is reduced during eccentric muscle activity, regardless of the degree of tension produced, highlighting the role of central inhibitory, protective mechanisms in the control of eccentric lengthening contractions. The protection against fatigue and the greater potentiation of power observed during the predominantly eccentric CM phase, compared to the push-off phase following anodal tsDCS is then worthy of further study as it may highlight differential modulatory actions on the inhibitory mechanisms proposed by Duchateau & Enoka [30] to be involved in the regulation of eccentric contractions and ultimately motor unit excitability.

Recent research investigating the effects of tcDCS on steady state cycling endurance found that anodal stimulation attenuated central fatigue mechanisms and allowed cyclists to increase their time to exhaustion [31]. Similar to the effects of tcDCS on supraspinal fatigue mechanisms, it appears that anodal tsDCS interferes with the regulatory actions that spinal circuits have on motor unit excitability and eccentric contraction fatigue. The neuromodulatory effects of direct current on central fatigue processes, highlight the need to account for, and perhaps exploit, these mechanisms in future neuromodulation studies.

The fatigue protection effects of anodal tsDCS on push-off performance may be also be explained by the permissive effects of tsDCS on CM performance. The likely effect of anodal tsDCS on eccentric inhibitory mechanisms and motor unit excitability would be unimpeded eccentric extensor lengthening [30]. This would increase downward velocity, displacement and muscle force development, and result in increased CM braking power, transition and push-off GRF, as the body is brought to a halt briefly prior to extending upwards from its lower position to take-off. Our findings post-anodal tsDCS are consistent with this observation. Improved coupling of body segment accelerations during countermovement may also contribute to the increase in these performance parameters and therefore cannot be discounted [15].

Although the mechanisms have not yet been fully elucidated, non-invasive anodal tsDCS can be predicted to lead to changes in spinal excitability via alterations in sensory axon excitability, inter-neuronal excitatory and inhibitory bias, and in communications between spinal cord segments [12]. Studies in humans [32] and animals [9, 10] suggest that the polarizing effects of direct current change the biophysical properties of the neural membranes, causing lasting changes in excitability. Depending on the current density and distribution within the tissues of the spinal cord, populations of interneurons will be affected differentially due to their spatial location, geometry and background level of activity [9, 10]. By studying a repeated whole body exercise, our observations point to a complex but positive interaction of tsDCS neuromodulatory effects. Our observations highlight the value of studying natural unconstrained functional motor tasks in contrast to more standard approaches, such as isometric muscle strength testing. The tsDCS effect is not uniform across the different phases of the VCJ and further investigations may allow us to further explore how alterations in spinal excitability affect motor control, motor unit recruitment and interlimb coordination during voluntary functional movement.

Individuals vary significantly in their response to tcDCS [33] and the participants in this study varied significantly in their responses to anodal tsDCS (Table 2). This may have been due to individual differences in somatotype, physical conditioning level and the level of effective current reaching neural compartments due to differences in body composition. However, an important genetic source of variation in response to direct current stimulation has recently been identified. A key mediator and promoter of activity-dependent neuroplasticity after physical activity [34–36] or direct current stimulation [37] is brain-derived neurotrophic factor (BDNF). Carriers of a common BDNF single nucleotide polymorphism (Met SNP; rs6265) have a reduced capacity for activity-dependent BDNF secretion [38] and have significantly different neuroplastic responses to tcDCS [37] and tsDCS [36] compared to those with the more common BDNF genotype. This may explain some of the unknown variability in our subjects and highlights the need to take account of such genetic factors as significant sources of variation in future neuromodulation studies [33].

This exploratory study was limited in that it was a preliminary observation study only. Further tsDCS investigation is merited using larger sample sizes to replicate these findings and elucidate the neurophysiological, biomechanical and genetic mechanisms underlying the lasting changes in neuromotor function. Further study would also help determine the potential neurorehabilitation benefits of anodal tsDCS for those with sporting injury or central nervous system injury that affects mobility, and possibly for falls prevention in the older adult. Indeed, the use of spinal rather than transcranial direct current stimulation, minimises the chances of unknown effects due to concurrent stimulation of brain areas unrelated to the targeted motor task. Studies including repeated tsDCS application with homogenous groups of well-trained endurance and power athletes are also merited. These may help elucidate more specific neuromuscular mechanisms underlying tsDCS effects and whether effects are additive or have a ceiling in performance gain. It should be noted that although none of our participants sustained any injury during this study, given the effect of tsDCS on protective fatigue mechanisms, the risk of injury in maximal eccentric exercise after tsDCS may be increased.

## Conclusion

In conclusion, using a double-blind, randomized, crossover sham-controlled study design, we have demonstrated for the first time that anodal tsDCS can induce lasting fatigue-resistance and maintain and enhance different aspects of whole body motor performance over time. Anodal tsDCS appears to have created a permissive state in spinal circuits where a lasting resistance to central fatigue was combined with varying degrees of multisegmental facilitatory, and neuroplastic effects to maintain and enhance different aspects of VCJ performance. The effects persisted for at least 3 hours and were achieved after one 15 application of easy to administer, comfortable anodal tsDCS stimulation and in the absence of a physical training intervention. These effects have immensely important implications for power endurance sport performance or sports in which there is high level of repeated reactive rebound activity. The lasting effects of anodal tsDCS on neuromotor circuits may also have important implications for falls prevention or rehabilitation after central nervous system injury.

## Supporting information

S1 Video Vertical countermovement jump performance. Performance outcomes were determined from the initiation of the jump until the point of take-off only.
(MP4)
